# The Use of Demoralization Scale in Italian Kidney Transplant Recipients

**DOI:** 10.3390/jcm9072119

**Published:** 2020-07-05

**Authors:** Yuri Battaglia, Luigi Zerbinati, Giulia Piazza, Elena Martino, Sara Massarenti, Michele Provenzano, Pasquale Esposito, Michele Andreucci, Alda Storari, Luigi Grassi

**Affiliations:** 1 Nephrology and Dialysis Unit, St. Anna University Hospital, 44124 Ferrara, Italy; a.storari@ospfe.it; 2 Department of Biomedical and Specialty Surgical Sciences, Institute of Psychiatry, University of Ferrara, 44124 Ferrara, Italy; zrblgu@unife.it (L.Z.); pzzgli@unife.it (G.P.); mrtlne1@unife.it (E.M.); msssra@unife.it (S.M.); luigi.grassi@unife.it (L.G.); 3 Nephrology and Dialysis Unit, Department of Health Sciences, Magna Graecia University, 88100 Catanzaro, Italy; michiprov@hotmail.it (M.P.); andreucci@unicz.it (M.A.); 4 Division of Nephrology, Dialysis and Transplantation, Department of Internal Medicine, University of Genoa and IRCCS Ospedale Policlinico San Martino, 16132 Genoa, Italy; pasqualeesposito@hotmail.com

**Keywords:** demoralization, psychiatric morbidity, kidney transplantation

## Abstract

Demoralization is a commonly observed syndrome in medically ill patients. The risk of demoralization may increase in patients after a kidney transplant (KTRs) because of the stressful nature of renal transplantation, psychosocial challenges, and adjustment needs. No study is available on demoralization amongst KTRs. The purpose of our study was to evaluate the validity of the Italian version of the Demoralization Scale (DS-IT) and the prevalence of demoralization in KTRs. Also, we aimed at exploring the association of the DS-IT with International Classification of Diseases (ICD) psychiatric diagnoses, post-traumatic growth (PTG), psychological and physical symptoms, and daily-life problems. A total of 134 KTRs were administered the MINI International Neuropsychiatric Interview 6.0. and the Diagnostic Criteria for Psychosomatic Research–Demoralization (DCPR/D) Interview. The DS-IT, the Edmonton Symptom Assessment System (ESAS), the Canadian Problem Checklist (CPC), were used to measure demoralization, physical and psychological symptoms, and daily-life problems; also, positive psychological experience of kidney transplantation was assessed with the PTG Inventory. Routine biochemistry and sociodemographic data were collected. Exploratory factor analysis demonstrated a four-dimensional factor structure of the DS-IT, explaining 55% of the variance (loss of meaning and purpose, disheartenment, dysphoria, and sense of failure). DS-IT Cronbach alpha coefficients indicated good or acceptable level of internal consistency. The area under the Receiving Operating Characteristics (ROC) curve for DS-IT (against the DCPR/D interview as a gold standard) was 0.92. The DS-IT optimal cut-off points were ≥20 (sensitivity 0.87, specificity 0.82). By examining the level of demoralization, 14.2%, 46.3%, 24.6%, and 14.6% of our sample were classified as having no, low, moderate, and high demoralization, respectively, with differences according to the ICD psychiatric diagnoses (*p* < 0.001). DS-IT Total and subscales scores were positively correlated with scores of ESAS symptoms and CPC score. A correlation between DS-IT loss of meaning and purpose subscale and PTGI appreciation of life subscale (*p* < 0.05) was found. This study shows, for the first time, a satisfactory level of reliability of the DS-IT and a high prevalence of severe demoralization in KTRs.

## 1. Introduction

The stressful nature of kidney transplantation has been well characterized in the literature [1]. The receipt of a new kidney may give rise to a new set of stressors, psychosocial challenges, and adaptive demands, which might increase the risk of any form of psychological distress and/or psychiatric disorders, especially anxiety and depression [2,3]. These syndromes, assessed by the International Classification of Diseases (ICD) or the Diagnostic and Statistical manual for Mental Disorders (DSM), can be diagnosed in about 20% of kidney transplant recipients (KTRs) [4,5].

Other significant clinical conditions (e.g., demoralization, health anxiety, and abnormal illness behavior), affecting a further 25–30% of KTRs, were detected by using the Diagnostic Criteria for Psychosomatic Research (DCPR) interview [6,7].

Among the DCPR diagnoses, demoralization has been found to affect about 20% of KTRs and to be associated with high levels of psychological and physical symptoms as measured by the Edmonton Symptom Assessment System (ESAS) [8].

Demoralization is a syndrome clinically separated from depression. It is characterized by a combination of distress and subjective incompetence, the loss of meaning and purpose in life, the lack of perceived social support, a sense of being trapped and personal failure, a cognitive attitude of pessimism, and hopelessness/helplessness [9,10,11]. Moreover, this psychological state has a significant role in negatively influencing a patient’s quality of life, coping styles, and dignity [12,13]. It is associated with suicide ideation [14,15,16], higher wish for hastened death [17,18], and a worsening of the prognosis [19,20,21] in patients affected by medical illness [11,22], cardiac transplant recipients [23,24], and KTRs [7].

In order to examine and screen for this condition, a specific psychometric tool, the Demoralization Scale (DS), has been developed [25]. Recent data have shown the validity of the DS when used in medical settings, including oncology, palliative care, and internal medicine [26,27,28], as well as psychiatry [29].

Since, to our knowledge, no data are available regarding the use of the DS in KTRs, the primary aim of this study was to extend the validity of the Italian version of the Demoralization Scale (DS-IT) and to evaluate the prevalence of demoralization in KTRs. The secondary aim was to explore any association between the DS-IT and psychiatric, psychosocial, and medical variables, specifically psychiatric diagnoses, post-traumatic growth, as well as physical and general problems or symptoms.

## 2. Materials and Methods

This study involved a consecutive series of prevalent KTRs who were followed up in the Nephrology Unit of the Ferrara University-Hospital. Inclusion criterion was a Karnofsky Performance Status Scale indicating a sufficient enough level of autonomy (score ≥50) [30]. Exclusion criterion was the absence of cognitive disorders (Mini Mental State Examination ≥24). The study was approved by the Local Institutional Review Board (approval code is 151297, on 17 March 2016). The procedures agreed with the Declaration of Helsinki and written informed consent was obtained from all the participants.

Members of the study population were approached during one of their routine follow-up nephrological visits in an index week and were met by the same expert psychiatrist, specialist in psychosomatic research. The monitoring, management, and treatment of KTRs were performed following the KDIGO guidelines [31].

Each patient was individually administered the Mini-International Neuropsychiatric Interview (MINI6.0) [32] and the DCPR interview [33], which both took about two hours. Before the diagnostic interviews, the patients filled in four self-report instruments: DS-IT [27], Post-Traumatic Growth Inventory (PTGI) [34,35], Edmonton Symptom Assessment System (ESAS-Revised) [8,36,37,38], and Canadian Problem Checklist (CPC) [39,40] (Table 1). In agreement with other screening programs [39], we administered together the ESAS and the Canadian Problem Checklist (CPC) within the COMPASS tool (Comprehensive Problem and Symptom Screening) [40].

The DCPR demoralization module (DCPR/D) is a semi-structured interview which allows making a diagnosis of demoralization if the following criteria are met: (1) feeling unable to cope with pressing problems and aware of having failed to meet his/her own expectations or those of others; (2) feeling helpless or hopeless or wanting to give up; (3) this mental state is prolonged and generalized (duration ≥ one month). The DCPR/D has been validated in several studies in medically ill patients [41,42].

As done in other studies, the level of demoralization was evaluated by using the DS-IT Total scores (low demoralization = <mean −1SD; moderate = mean −1SD to mean +1SD; and high = >mean +1SD) [43].

Sociodemographic and clinical data, including immunosuppressive agents, were collected, and routine biochemistry, using standard auto analyzer techniques, was repeated. These data were used for final statistical analysis.

### Statistical Analysis

Statistical analysis was performed using the SPSS-26 package and the level of statistical significance was set at *p* < 0.05. Continuous variables were reported as either mean ± standard deviation (SD) or median and interquartile range (IQR) based on their distribution. Categorical variables were shown as frequencies (%).

An exploratory factor analysis, using the principal factor method with orthogonal varimax rotation, was used to investigate the DS-IT structure [27]. We computed the factor loadings as part of the outcome from factor analysis, which serves as a data reduction method designed to explain the correlations between the items using a smaller number of factors, which indicate the relative importance (or “magnitude”) of the items composing the DS-IT with their relationship between the items and the main underlying factors (expressed in values 0–1). In the original English validation study of the DS [25], five factors were shown (named loss of meaning/purpose, dysphoria, disheartenment, helplessness, and sense of failure). The Kaiser–Meyer–Olkin test was performed to confirm the suitability for factor analysis (>0.8 indicates adequate sampling) [44]. Internal consistency of each scale was estimated by calculating Cronbach coefficient α.

Receiving Operating Characteristics (ROC) analysis was used to explore the optimal DS-IT cut-off score in detecting clinical cases in our sample. It was employed to determine the ability of DS-IT to detect demoralization cases defined by using DCPR/D (caseness). Combined sensitivities and specificities were visualized in the ROC curve. The Area Under the Curve (AUC) provides an estimate of overall discrimination for evaluations of possible adequate cut-off value of the DS-IT. The AUC of 0.5–0.7 indicates low accuracy, 0.7–0.9 indicates moderate accuracy, and 0.9–1.0 indicates high accuracy [45]. The Youden index (J) was computed to find a cut-point that maximizes the variable’s differentiating ability from the ROC curves. J is defined as the variable’s value for which equal weight is given to sensitivity and specificity [46].

T-test, chi-square test, and ANOVA were employed to determine the relationship of degree of demoralization and PTGI with ICD-10 diagnoses and clinical variables. The correlation among DS-IT score, ESAS scores, PTGI score, and sociodemographic characteristics was assessed by Pearson’s correlation coefficient for parametric data and Spearman correlation coefficient for nonparametric data.

## 3. Results

### 3.1. Characteristics of the Sample and Prevalence of Demoralization

Data pertaining to 134 out of 145 consecutive KTR outpatients were collected. Two patients were excluded, and nine patients declined to participate (six for work or family reasons and three because of health reasons). The detailed sociodemographic and clinical characteristics of the sample are shown in Table 2.

In summary, 67.2% were men and the mean age was 56.1 (SD 12) years. Forty-six (34.3%) patients reported an ICD-10 psychiatric diagnosis, of which adjustment disorders (*n* = 21, 45.6% of all psychiatric diagnosis and 15.6% of the whole sample) were the most prevalent. Eighty-three KTRs (62%) were on triple immunosuppressant medications, specifically steroids (84.3%), calcineurin inhibitors (90,3%), mycophenolate (67.7%), mTOR inhibitors (8.3%), azathioprine (10.4%).

The distribution of the DCPR and ICD diagnoses are presented in Table 3. Of the total sample, 85 patients (63.4%) presented symptoms meeting the criteria for at least one DCPR diagnosis, with 43 subjects (32.1%) reporting one DCPR diagnosis (DCPR = 1) and 42 (31.3%) more than one (DCPR > 1). According to the DCPR/D, 23 patients (17.2%) resulted in positive “cases” for demoralization.

### 3.2. Factor Structure and Internal Consistency of the DS-IT

The Kaiser–Meyer–Olkin measure of sample adequacy was 0.85, indicating that the factor analysis was appropriate. Principal component analysis (varimax rotation with Kaiser normalization) identified four factors, which explained 55% of the variance. These factors were loss of meaning and purpose, disheartenment, dysphoria, and sense of failure (Table 4).

Loss of meaning and purpose explained 15.1% of the variance and consisted of eight items, comprising the four items from the original subscale (items 3, 4, 7, 14, and 20) as well as three (items 8, 9, and 23) items belonging originally to the disheartenment scale. Disheartenment explained 21.1% of the variance and included nine items: five of them belonging to the original disheartenment scale (items 5, 18, 21, 22, and 24), plus item 2 belonging to the original loss of meaning scale, plus two items (6 and 12) belonging to the original sense of failure scale, plus item 13 belonging to the original dysphoria scale. Dysphoria explained 10.6% of the variance and included four items which were identical with the original subscale (items 10, 11, 15, and 16). Sense of failure explained 8.3% of the variance and consisted of three items of the original scale (items 1, 17, and 19). All Cronbach alpha coefficients of the DS-IT indicated good or acceptable level of internal consistency and had the following values: loss of meaning and purpose α = 0.84; disheartenment α = 0.89; dysphoria α = 0.72; and sense of failure α = 0.66. Cronbach alpha for DS-IT Total was α = 0.74.

### 3.3. DS-IT Cut-Off Scores and Relationship with the ICD Diagnoses

**Figure 1:**
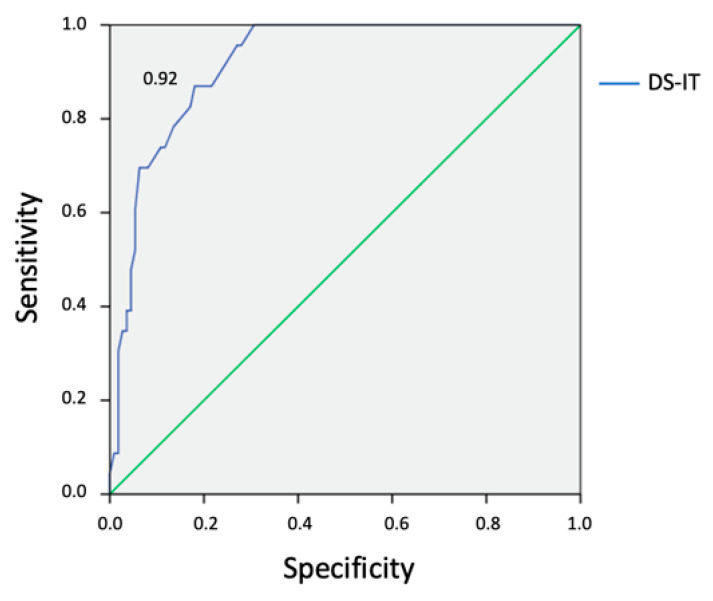
Receiver operating characteristic analysis of caseness on Diagnostic Criteria for Psychosomatic Research Interview—Demoralization section is illustrated. DS-IT: Demoralization Scale–Italian Version.

By conducting ROC analysis (DS-IT vs. DCPR/D as gold standard), the area under the ROC curve for the DS-IT was 0.92 (95% CI = 0.87–0.97) (Figure 1). The ROC curve showed relatively moderate accuracies of the DS-IT to detect DCPR/D caseness. The DS-IT cut-off point ≥ 20 optimized sensitivity (87%) and specificity (82%) for caseness. By using this score, 38 patients (28.4%) were demoralized (DS-IT cases), of whom 19 had DCPR/D diagnosis. Only four DCPR/D cases were not DS-IT cases.

According to the methodology used by Mullane [43], 19 (14.2%), 62 (46.3%), 33 (24.6%), and 2 (14.9%) were classified as having no, low, moderate, and high demoralization (Mullane cases), respectively. Thirty-eight (28%) Mullane cases did exceed the DS-IT cut-off and had moderate or high demoralization while only 1 (4.3%) DCPR/D case had a low level of demoralization. All (19) Mullane no-cases resulted in being DS-IT non-cases or DCPR/D non-cases. The level of demoralization varied according to the ICD diagnoses (*p* < 0.001) (Table 5).

Regarding ICD-10 psychiatric diagnosis (as assessed through the MINI6.0 interview), among those who did not show any psychiatric disorder, 18/88 patients (20.4%) had no demoralization, 58/88 patients (65.9%) low demoralization, 11/88 moderate (12.5%) demoralization, and 1/88 patient high (1.2%) demoralization. When examining those with a current ICD psychiatric diagnosis (46/134, 34.2%), 13/14 (92.8%) patients with anxiety disorders, 18/21 patients (85.7%) with adjustment disorders, and 10/11 (90.9%) with depressive disorders were moderately to severely demoralized (comparison between patients without and with a psychiatric diagnosis on the grade of demoralization: chi-square 75.6, *p* < 0.01). Globally, 112/115 of those who showed from low to high levels of demoralization did not satisfy the ICD criteria for depressive disorders.

### 3.4. Relationship of the DS-IT with PTG level, ESAS, CPC, and Sociodemographic Variables

The mean PTGI score was 52.02 (SD 20.69). Women (57.2 SD 23.1) had higher scores of PTGI than men (49.5 SD 19.0) (F 4.2, *p* < 0.05). Sociodemographic (education, marital status, housing, occupation) and clinical characteristics (psychiatric diagnosis ICD and nephropathy) were not significantly associated with PTGI. A negative correlation between DS loss of meaning and purpose subscale and PTGI appreciation of life subscale (*p* < 0.05) was found (Table 6).

Total and subscale DS-IT scores were positively correlated with ESAS symptoms, ESAS sub-scores, and ESAS Global Distress score, as well as CPC score (Table 7).

No significant differences of DS-IT total and subscales score was found with sociodemographic characteristics (age, sex, housing, employment, nephropathy), excepted for marital status (*p* = 0.05). Widows (29.0 SD 22.7), followed by divorced (18.7 SD 12.4) and single (15.8 SD 16.8), had higher DS-IT total scoring compared to married individuals (13.0 SD 11.9).

## 4. Discussion

In this study, we analyzed, for the first time, the application of the Italian version of the DS to assess the demoralization among patients who underwent kidney transplantation.

The results of exploratory factor analysis demonstrated a four-dimensional factor structure of the DS-IT, namely loss of meaning and purpose, disheartenment, dysphoria, and sense of failure, in our sample, explaining 55% of the variance. These findings are in line with a previous validation study of the DS-IT, carried out in Italian patients affected by different forms of cancer, which found four factors [27] showing good internal consistency levels for the global scale and four subscales. However, our four factors revealed slightly different item clusters compared with the previous studies [25,27,43]. Indeed, while dysphoria and sense of failure subscales consisted of the items of the original version of the DS-IT, the factors loss of meaning and disheartenment also consisted of items from other factors. The different loading of some items with respect to the original factors could be due to the spread of items across the dimensional nature of demoralization and maybe cultural influences [27]. Cronbach α coefficients ranged between 0.74 and 0.84 for the single and total score, indicating an acceptable level of internal consistency.

Regarding the general level of demoralization (DS-IT total), we found a lower score (14.64, SD 13.84) than that reported in cancer patients and palliative care settings which ranged from 19.9 (SD 14.6) to 61.3 (SD 12.4). One possible reason could be related to the better physical and medical conditions of our sample, considering that demoralization has been reported to be higher among patients with lower performance levels or advanced stages of illness [47]. Also, it is possible that the ability to project oneself in the future after kidney transplant can increase the sense of purpose and meaning in life, in comparison with patients with life-threatening disease or at the end of life [48]. Also, socioeconomic reasons, such as the low rate of unemployment in our population (7.5%), can play a significant role, as shown by the fact that demoralization has also been shown to be associated with lack of work [41]. When analyzing the marital status, a higher score in singles (15.83 SD 16.88) than married KTRs (13.01 SD 11.94) (F 2.369, *p* < 0.05) was found. These findings are in agreement with the reported associations of demoralization in heart transplant patients [24] and in other medical settings [49,50], confirming the role of partners’ support of patients in coping with life problems.

When investigating the prevalence of demoralization, we used two different methods to assess cases, namely Mullane’s method and a case-rule system based on cut-off scoring (DS-IT Total ≥ 20), assuming the DCPR/D diagnosis as the “gold-standard” tool reference to detect cases [7]. On one hand, applying a cut-off score of ≥ 20, our results showed a tendency to overestimate caseness, with a prevalence of DS-IT cases higher by 11.2% than that of DCPR/D caseness. However, six DS-IT cases had high demoralization without an DCPR/D diagnosis, and only four DCPR/D cases had a total score lower than the DS-IT score with low or moderate demoralization. With respect to this finding, the DS-IT was also able to identify severe forms of demoralization, requiring psychiatric care, among DCPR/D non-cases, setting aside slightly or moderately demoralized KTRs, with a debatable relevance in clinical practice. On the other hand, following the methodology of Mullane, almost all (85.8%) KTRs reported demoralization feelings, of whom only 14.9% had a high level of demoralization. Taking into account only these data on severe demoralization, they are consistent with those ones observed with the DCPR diagnosis (17.2%) and also other studies in palliative care settings in Italy (17%), Germany (15.7%), and Ireland (14%). In contrast with these data, our KTRs severely demoralized were just over half of those reported for heart transplantation [24].

In the second step of our analysis, we showed that almost all KTRs with a diagnosis of depressive disorder were also suffering from severe demoralization, while different distributions had been found regarding other ICD diagnoses and levels of demoralization. Besides, most of the KTRs, who received an ICD psychiatric diagnosis, were affected by a state of demoralization, especially of low intensity according to Mullane’s indications. These findings support previous studies carried out in cardiac transplant and medically ill patients [24,42], showing that demoralization is a different construct to depression, although some overlap with depression is more evident when the severity of demoralization increases. It also has to be underlined that demoralization was not necessarily related to a formal current psychiatric diagnosis. In fact, a consistent number of KTRs who did not receive any psychiatric diagnosis had low (65.9%) or moderate (12.5%) demoralization. This confirms the above-mentioned studies [23,24] and strongly suggests the need to properly assess demoralization in all patients.

Regarding the relationship of demoralization with psychological and physical dimensions and daily-life problems, as assessed through the COMPASS, we observed a positive association of the level of demoralization with symptom burden (ESAS psychological and physical) and several life problems (CPC), endorsing that a range of factors including poorly controlled physical symptoms, inadequately treated depression, and anxiety [28] may determine an erosion of the sense of purpose and meaning of life, disheartenment, and helplessness/hopelessness feelings. In terms of explanation and the need for further exploration, many factors, such as being dependent on results of laboratory tests and diagnostic procedures, worries and concerns related to medical complications (e.g., kidney allograft rejection, immunosuppressive therapy effects), and their future (e.g., need to return to dialysis treatment), could facilitate the development of demoralization in KTRs.

Interestingly, when examining the relationship between demoralization and post-traumatic growth, we found an inverse correlation between the DS-IT “loss of meaning and purpose” factor and the PTGI “appreciation of life” subscale. This finding suggests that there are aspects of these two constructs that are part of an overlapping dimension, as also found in another study [51] carried out in cancer patients. Furthermore, “sense searching” showed to have different effects on demoralization and post-traumatic growth. The interaction of seeking sense with the recognition of benefits might predict demoralization. Conversely, subjects with more sense and perceived benefit generally experience less demoralization [34]. In other words, these results support the importance of giving a meaning to the motivations that lead patients to kidney transplantation. This awareness could protect the patients after kidney transplant from the loss of appreciation of life and the state of demoralization.

The strength of this study is that it evaluated, for the first time, the validity of the DS-IT to assess the Demoralization Syndrome and its correlation with the other measure of demoralization, DCPR/D. Although the DS-IT was used in patients affected by end-of-life and/or progressive medical disease [28], it has not been proven in KTRs.

There are, however, limitations in our study that also should be mentioned. First of all, the small sample size of our study population does not allow us to generalize our results. Thus, further multicenter studies on larges samples of KTRs should be considered for future research. A second limitation of our study is that there was no control group of patients with other kidney conditions, including in-patient units and in-patients with other renal disorders, that could have given us more details about the rate and characteristics of psychosocial morbidity in other areas of nephrology. Future studies should examine this specific area considering that it is well described that dialysis patient populations are subject to significant mental and medical stress, and a comparison of demoralization before and after transplant (with the patients serving as their own controls) or with a dialysis arm may help to understand if transplant significantly reduces the incidence of demoralization.

In addition, we did not evaluate the effect of vitamin D on demoralization, although low vitamin D concentration is associated with mood disorders in KTRs [52,53]. Also, we did not take into account the level of physical activity, a relevant variable which can improve mood and reduce anxiety [54,55,56]. Furthermore, when conducting our study, a shorter version of the DS [57] was not available yet. This could be the aim of future studies to assess the validity of this as a new screening tool for demoralization in clinical settings.

In conclusion, although further studies are necessary to better explore the presence of demoralization and its implications in terms of quality of life and prognosis, as done in other medical settings, our findings highlighted the importance of detecting this construct in KTRs. In this regard this study provides data showing a satisfactory level of validity and reliability of a scale, the DS-IT, that could be used in nephrology to assess demoralization amongst KTRs. Further research is also required to assess the efficacy of psychosocial interventions, which have been used to improve demoralization in medically ill patients and that could increase KTRs’ sense of purpose and meaning, and decrease their sense of suffering.

## Figures and Tables

**Table 1:** Features of interviews and self-report instruments used in the study.

Tool	Characteristics
**Interview**	
Mini-International Neuropsychiatric (MINI6.0) [32]	It is a short, structured diagnostic interview that has been validated against both the Structured Clinical Interview for DSM diagnoses (SCID-P) and the Composite International Diagnostic Interview (CIDI) for ICD-10 diagnoses in different countries, including Italy.
Diagnostic Criteria for Psychosomatic Research (DCPR) [33]	It investigates a set of 12 syndromes organized in 3 different clusters: abnormal illness behavior (i.e., disease phobia, thanatophobia, health anxiety, illness denial); somatization and its different expressions (i.e., persistent somatization, functional somatic symptoms secondary to a psychiatric disorder, conversion symptoms, anniversary reaction); irritability (i.e., irritable mood and type A behavior), and other relevant clinical constructs (i.e., demoralization and alexithymia).
**Self-report instruments**	
Demoralization Scale (DS-IT) [27]	It is used in its 24-item format, each item ranged on 5-point Likert scale (0 = never; 4 = all the time) over the past 2 weeks. It is divided in four subscales: loss of meaning and purpose, dysphoria, disheartenment, and sense of failure. A total score is obtained by summing up the single subscales scores with a maximum score of 96.
Post-Traumatic Growth Inventory (PTGI) [34,35]	It evaluates the positive changes experienced in the aftermath of a traumatic event. It consists of 21 items divided in five factors: New Possibilities, Relating to Others, Personal Strength, Spiritual Change, and Appreciation of Life. Each item scored on 6-point Likert scale (0 = I did not experience this change as a result of my crisis; 5 = I experienced this change to a very great degree as a result of my crisis).
Edmonton Symptom Assessment System (ESAS-Revised) [8,36,37,38]	It examines the severity of physical (i.e., pain, tiredness, nausea, drowsiness, lack of appetite, shortness of breath) and psychological symptoms (i.e., depression, anxiety, feeling of not well-being) on a 0 (no symptom) to 10 (the worst symptom) scale. An optional 10th psychological symptom was also added in this study, specifically the emotional distress item which corresponds to the Distress Thermometer, a worldwide validated tool to measure distress also on a 0–10 Visual Analog Scale. The physical distress sub-score (ESAS-PHYS) is the sum of scores for the six physical symptoms, while the psychological distress sub-score (ESAS-PSY) is the sum of the scores of the four psychological symptoms. A Global ESAS-TOT score is given by summing up all the scores on the single ESAS symptoms.
Canadian Problem Checklist (CPC) [39,40]	It consists of a list of 21 problems each rated in a yes/no (0–1) format and grouped into 6 categories: practical, social/family, emotional, spiritual, informational, and physical problems.

**Table 2:** Sociodemographic and clinical variables of the sample.

Clinical Variables		Sociodemographic Variables	
Age, years *	56.1 ± 12.0	Education, years *	11.5 ± 4.5
Males, *n* (%)	90 (67.2)	*Occupation*	
Kidney graft months, median (IQR)	85 (34–178)	Laborer, *n* (%)	15 (11.2)
Race-Caucasian, *n* (%)	126 (94)	Office Worker, *n* (%)	27 (20.1)
		Freelance Professional, *n* (%)	5 (3.7)
*Cause of CKD*		Other Jobs, *n* (%)	15 (11.2)
Glomerulonephritis, *n* (%)	55 (41)	Housewives, *n* (%)	3 (2.2)
ADPKD, n (%)	25 (18.7)	Retired, *n* (%)	59 (44.1)
Diabetes Mellitus, *n* (%)	6 (4.5)	Unemployed, *n* (%)	8 (6.0)
Hypertension, *n* (%)	4 (3)	Unemployable, *n* (%)	2 (1.5)
Other, *n* (%)	44 (32.8)		
		*Living Situation*	
*Blood Test Values*		Family, *n* (%)	93 (69.4)
Creatinine, mg/dL *	1.4 ± 0.5	Parents, *n* (%)	22 (16.4)
GFR-MDRD, mL/min *	53.2 ± 17.5	Alone, *n* (%)	11 (8.2)
Hemoglobin, g/dL *	12.4 ± 1.5	Others, *n* (%)	8 (5.9)
		*Marital Status*	
		Single, *n* (%)	29 (21.5)
		Married, *n* (%)	89 (66)
		Divorced, *n* (%)	10 (7.5)

* Data are expressed as mean ± standard deviation; ADPKD: Autosomal Dominant Polycystic Kidney Disease; CKD: Chronic Kidney Disease; GFR-MDRD: glomerular filtration rate according to the equation from the Modification of Diet in Renal Disease Study; IQR: Interquartile Range; PTH: Parathormone.

**Table 3:** Ranking order of DCPR and ICD diagnoses.

Rank ICD Diagnosis		DCPR Diagnosis	
No diagnosis, n (%)	88 (65.7)	No diagnosis, n (%)	49 (36.5)
Reaction to severe stress and adjustment disorders, n (%)	21 (15.7)	Cluster AIB, n (%)	43 (31.3)
		Cluster Irritability, n (%)	42 (31.3)
Anxiety disorders, n (%)	14 (10.4)	Cluster Somatization, n (%)	26 (19.3)
Mood [affective] disorders, n (%)	11 (8.2)	Alexithymia, n (%)	31 (23.1)
		Demoralization, n (%)	23 (17.2)

AIB: Abnormal Illness Behavior; DCPR: Diagnostic Criteria for Psychosomatic Research; ICD: International Classification of Diseases.

**Table 4:** Item and scale characteristics (principal component analysis, varimax-rotated 4-factor solution) of the Italian version of the Demoralization Scale in KTRs.

Dimensions and Items	Factor Loadings			
	F1	F2	F3	F4
*Disheartenment (explained variance, 21.1%)*				
21. I feel sad and miserable	0.733			
6. I am in good spirits *	0.714			
18. I feel distressed about what is happening to me	0.694			
24. I feel trapped by what is happening to me	0.668			
5. I no longer feel emotionally in control	0.636			
2. My life seems to be pointless	0.630	0.514		
22. I feel discouraged about life	0.624			
13. I have a lot of regret about my life	0.592			
12. I cope fairly well with life *	0.578			
*Loss of meaning and purpose (explained variance, 15.1%)*				
20. I would rather not be alive		0.678		
4. My role in life has been lost		0.673		
3. I suffer great anxiety about it	0.521	0.665		
9. I feel hopeless		0.625		
14. Life is no longer worth living		0.552		
23. I feel quite isolated or alone		0.538		
8. I feel that I cannot help myself		0.510	0.454	
7. No one can help me		0.425		
*Dysphoria (explained variance, 10.6%)*				
10. I feel guilty			0.811	
11. I feel irritable			0.782	
16. I am angry about a lot of things			0.771	
15. I tend to feel hurt easily			0.604	
*Sense of failure (explained variance, 8.3%)*				
19. I am a worthwhile person *				0.788
1. There is a lot of value in what I can offer others *				0.781
17. I am proud of my accomplishments *				0.616

* Data indicate reverse items.

**Table 5:** Level of demoralization among ICD-10 syndromes.

Demoralization		Rank ICD diagnosis				
		No Diagnosis	Anxiety Disorders	Mood [Affective] Disorders	Reaction to Severe Stress and Adjustment Disorders	Total
No	n	18	0	1	0	19
	%	13.4	0	0.7	0	14.2
Low	n	58	1	0	3	62
	%	43.3	0.7	0	2.2	46.3
Moderate	n	11	9	4	9	33
	%	8.2	6.7	3.0	6.7	24.6
High	n	1	4	6	9	20
	%	0.7	3	4.5	6.7	14.9
Total	n	88	14	11	21	134
	%	65.7	10.4	8.2	15.7	100

ICD: International Classification of Diseases.

**Table 6:** Correlation between DS-IT subscales and PTGI subscales.

	DS-IT Subscales			
PTGI Subscales	Disheartenment	Sense of Failure	Dysphoria	Loss of Meaning and Purpose
Relating to Others	0.053	0.009	−0.033	−0.074
	0.271	0.458	0.354	0.196
New Possibilities	−0.029	−0.016	0.022	−0.111
	0.369	0.429	0.400	0.101
Personal Strength	0.010	0.078	−0.003	−0.121
	0.456	0.184	0.488	0.082
Spiritual Change	0.022	−0.004	−0.037	−0.032
	0.400	0.483	0.334	0.356
Appreciation of Life	−0.089	−0.047	−0.031	−0.143 *
	0.153	0.296	0.362	0.049

* *p* < 0.05; DS-IT: Demoralization Scale–Italian Version; PTGI: Post-Traumatic Growth Inventory.

**Table 7:** Correlation between DS-IT and scores of ESAS.

ESAS	DS-IT				
	Total	Disheartenment	Loss of Meaning and Purpose	Dysphoria	Sense of Failure
Pain	0.194	0.175	0.150	0.188	0.132
	*p* = 0.024	*p* = 0.043	*p* = 0.085	*p* = 0.030	*p* = 0.129
Tiredness	0.315	0.260	0.243	0.293	0.284
	*p* < 0.001	*p* = 0.002	*p* = 0.005	*p* = 0.001	*p* = 0.001
Nausea	0.219	0.210	0.184	0.237	0.066
	*p* = 0.011	*p* = 0.015	*p* = 0.034	*p* = 0.006	*p* = 0.447
Depression	0.702	0.703	0.639	0.528	0.421
	*p* < 0.001	*p* < 0.001	*p* < 0.001	*p* < 0.001	*p* < 0.001
Anxiety	0.557	0.532	0.501	0.455	0.346
	*p* < 0.001	*p* < 0.001	*p* < 0.001	*p* < 0.001	*p* < 0.001
Drowsiness	0.440	0.412	0.341	0.417	0.275
	*p* < 0.001	*p* < 0.001	*p* < 0.001	*p* < 0.001	*p* = 0.001
Lack of appetite	0.189	0.173	0.117	0.211	0.112
	*p* = 0.029	*p* = 0.045	*p* = 0.178	*p* = 0.014	*p* = 0.197
Feeling of not well-being	0.521	0.492	0.323	0.489	0.415
	*p* < 0.001	*p* < 0.001	*p* < 0.001	*p* < 0.001	*p* < 0.001
Shortness of breath	0.100	0.077	0.126	0.118	0.010
	*p* = 0.250	*p* = 0.378	*p* = 0.146	*p* = 0.174	*p* = 0.910
Stress	0.479	0.394	0.401	0.504	0.312
	*p* < 0.001	*p* < 0.001	*p* < 0.001	*p* < 0.001	*p* < 0.001
ESAS-PSY	0.720	0.673	0.599	0.633	0.474
	*p* < 0.001	*p* < 0.001	*p* < 0.001	*p* < 0.001	*p* < 0.001
ESAS-PHYS	0.432	0.385	0.341	0.427	0.276
	*p* < 0.001	*p* < 0.001	*p* < 0.001	*p* < 0.001	*p* = 0.001
ESAS-Total	0.650	0.597	0.530	0.599	0.423
	*p* < 0.001	*p* < 0.001	*p* < 0.001	*p* < 0.001	*p* < 0.001
CPC Total	0.482	0.403	0.567	0.495	0.482
	*p* < 0.001	*p* < 0.001	*p* < 0.001	*p* < 0.001	*p* < 0.001

CPC: Canadian Problem Checklist; DS-IT: Demoralization Scale–Italian Version; ESAS: Edmonton Symptom Assessment System; ESAS-PHYS: physical distress sub-score; ESAS-PSY: psychological distress sub-score; ESAS-Total: global distress score.

## Abbreviations

CPC	Canadian Problem Checklist
DCPR	Diagnostic Criteria for Psychosomatic Research
DSM	Diagnostic and Statistical Manual of Mental Disorders
ESAS	Edmonton Symptom Assessment System
ICD	International Classification of Diseases
KTRs	kidney transplant recipients
MINI	Mini-International Neuropsychiatric Interview
DS-IT	Italian Version of Demoralization Scale
PTGI	Post-Traumatic Growth Inventory
